# Acetonitrile-assisted exfoliation of layered grey and black arsenic: contrasting properties†

**DOI:** 10.1039/c9na00754g

**Published:** 2020-02-03

**Authors:** Nikolas Antonatos, Vlastimil Mazánek, Petr Lazar, Jiri Sturala, Zdeněk Sofer

**Affiliations:** Department of Inorganic Chemistry, University of Chemistry and Technology Prague Technická 5, 166 28 Prague 6 Czech Republic zdenek.sofer@vscht.cz; Regional Centre of Advanced Technologies and Materials, Faculty of Science, Palacký University Olomouc tř. 17. listopadu 12 77 146 Olomouc Czech Republic

## Abstract

In recent years, two-dimensional monoelemental nanostructures beyond graphene have received great attention due to their outstanding properties. Out of these elements, only arsenic is known to form different allotropes with a layered structure in the bulk form. Orthorhombic arsenic, also termed “black arsenic”, is a metastable form of arsenic with a structure analogous to that of black phosphorus and rhombohedral arsenic is known as “grey arsenic”. Here, we compare the exfoliation of black and grey arsenic in acetonitrile in high yield forming stable colloidal solutions of exfoliated materials. Together with the exfoliation procedure, detailed structural and chemical analyses are provided and potential applications in gas sensing and photothermal absorption are demonstrated for potential future arsenic-based devices.

## Abstract 2

We report herein the successful exfoliation of grey and black arsenic through shear force in acetonitrile. Both exfoliated materials displayed great photothermic effect while black arsenic was shown to be a better candidate for impedimetric volatile organic compounds sensor.

## Graphical abstract

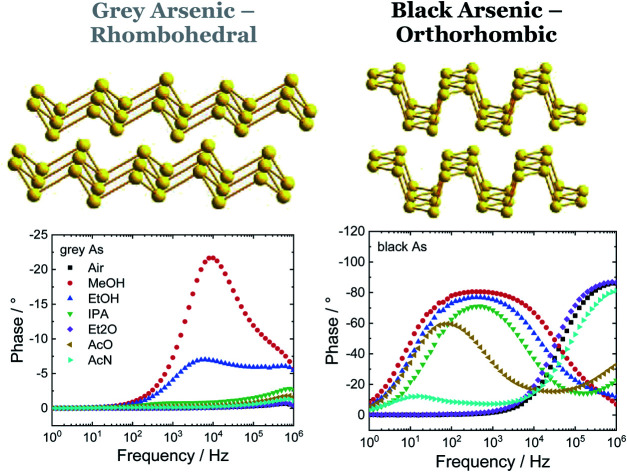

## Introduction

In recent years, two-dimensional (2D) layered materials, such as graphene,^1–3^ hexagonal boron nitride (h-BN),^4,5^ transition metal dichalcogenides (TMDs)^6–9^ and monoelemental 2D materials like black phosphorus^10–12^ have attracted significant attention due to their unique properties and large application potential. There has been extensive research in the field of multi-elemental 2D materials, and currently monoelemental 2D materials beyond graphene have re-emerged in the vanguard of materials research.^13^ While graphene, silicene, germanene and phosphorene have been extensively studied in recent years,^12,14^ arsenene, antimonene and bismuthene have been mostly explored using theoretical calculations.^14–20^ Especially for arsenene and its experimentally synthetic methods, there have been a limited number of reports.

**Fig. 1:**
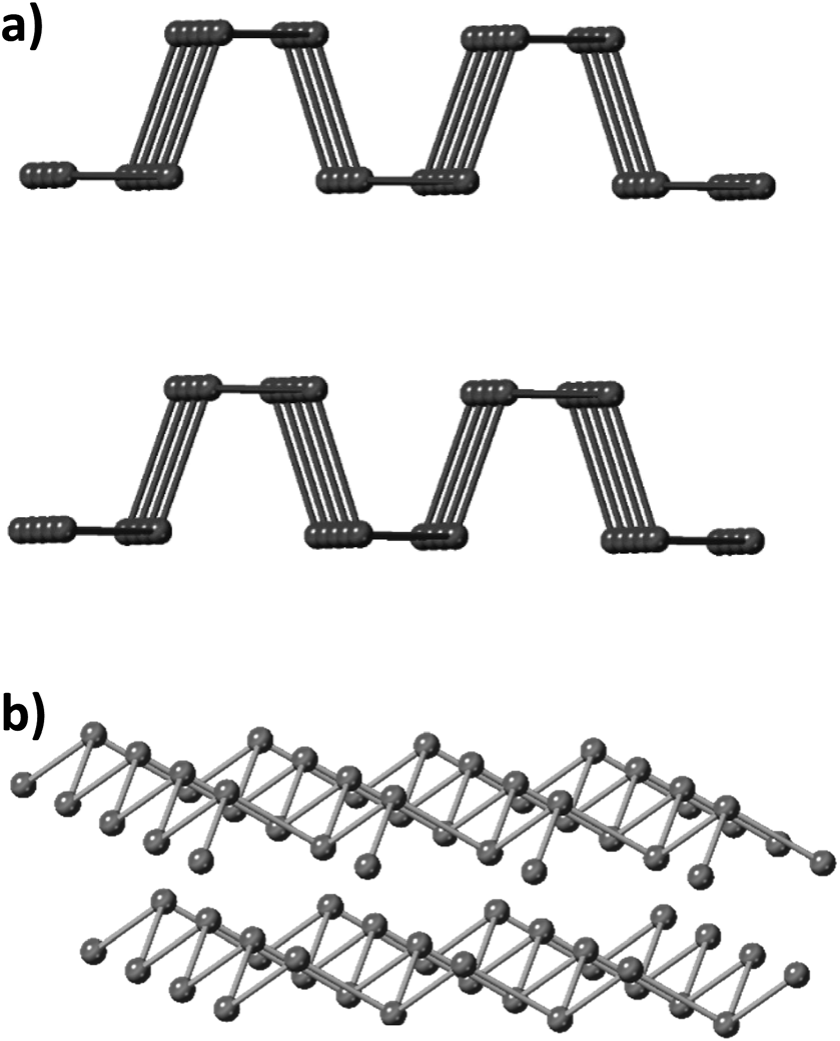
(a) Orthorhombic crystal structure and (b) rhombohedral crystal structure. The first crystal structure is the thermodynamically stable form of phosphorus at room temperature and normal pressure while the rhombohedral structure is the most common for antimony, bismuth and arsenic.

The group V, also called pnictogens, elemental layered materials have been characterized as promising 2D materials with semiconducting properties.^21,22^ Black phosphorus (BP) holds an orthorhombic crystalline form and the P atoms are positioned into hexagonal puckered layers held together by van der Waals forces.^23^ This structural allotrope is the thermodynamically most stable form of phosphorus, however its kinetic barrier makes it synthesis difficult and challenging (Fig. 1a). On the other hand, heavier pnictogens, namely As, Sb and Bi, crystallize into a rhombohedral crystal structure which is their most thermodynamically stable allotropic form (Fig. 1b), despite the existence of other allotropes including the true van der Waals layer orthorhombic form.^22^

Arsenic (As) has been widely known as a highly toxic material, but it is essential for human life requiring up to 25 μg per day.^24^ Furthermore, arsenic is mainly employed in the car industry for strengthening the lead components of car batteries^25^ and it is a common n-type dopant found in a wide range of semiconducting materials.^26^

At ambient pressures, arsenic is a covalently bonded compound existing in the rhombohedral (A7) phase called grey arsenic (g-As). In addition, arsenic can be found in two more allotropes: yellow arsenic (y-As) and black arsenic (b-As). y-As comprises of tetrahedral As_4_ molecules similar to white phosphorus (P_4_),^27^ while b-As crystallizes in an orthorhombic structure isostructural to black phosphorus (BP).^28^ A single layer of black arsenic, b-arsenene, has been theoretically calculated to possess a large bandgap,^29^ which in combination with its low thermal conductivity^30^ render it as a promising material for thermoelectric applications^31^ and vapor sensing.^32^ Thus, black arsenic has been termed as the “cousin” of black phosphorus.

The rhombohedral A7 structure is characteristic of heavy pnictogens which are referred to as grey arsenic, grey antimony and metallic bismuth. It is a low symmetry, three-fold coordinated, and layered structure. The layers are stacked in the (111) direction and consist of puckered, six-membered rings of coordinated atoms in a similar fashion to carbon atoms in graphene sheets.^33^ The differences between in-plane and out-of-plane interatomic distances are adequately high to provide the layered structure with an anisotropy of the physical properties. Subjected to sufficiently high pressures, arsenic undergoes a transition from a semi-metallic A7 to a metallic simple cubic structure.^34^ Despite the layered nature of A7, there are notable interlayer interactions of arsenic orbitals across layers leading to partially covalent bonds in the out-of-plane direction. This has a significant effect on the properties of the material especially in performance of the exfoliation procedure, where a lower degree of anisotropy can lead to a lateral size reduction of the exfoliated materials.^11^

On the other hand, the orthorhombic structure of b-As is composed of puckered layers held together by weak van der Waals forces similar to graphite and black phosphorus.^35^ This structure is unique for the structural anisotropy inside its layers, which is very rare among 2D materials. Hence, the anisotropy in the physical properties may lead to remarkable device applications which are not possible with isotropic materials.

Shear force exfoliation of grey arsenic has already been demonstrated through the use of kitchen blenders^22^ and in aqueous surfactant sodium cholate^36^ and more recently in various organic solvents.^37^ Herein, we report an effective acetonitrile-assisted exfoliation procedure for black and grey arsenic forming a stable dispersion in acetonitrile. Acetonitrile has been chosen as the exfoliating solvent because it has been used previously for the successful exfoliation of black phosphorus, it has a low boiling point, it can keep a stable dispersion of the exfoliated flakes, prevent the degradation of the exfoliated sheets due to oxidation and can be easily removed without leaving any impurities.^38^

Both exfoliated materials were characterized in detail showing the presence of single and few layer sheets. The exfoliated arsenene samples in both allotropic modifications were used for gas sensing applications and photothermal absorption experiments. These results will open up new horizons for the fabrication of arsenic-based nanodevices.

## Experimental procedure

### Materials

Grey (rhombohedral) arsenic was obtained from Alfa Aesar (99.9999%). Black (orthorhombic) arsenic was used in its natural form (Arsenolamprite; Copiapó, Atacama Desert, Chile) of sheet crystals with a 5–10 mm size. Both arsenic allotropes were ground in an agate mortar and sieved to separate particles below 0.5 mm. High-energy shear force exfoliation was performed in dry deoxygenated acetonitrile under an argon atmosphere using 100 mL of acetonitrile and 1 g of arsenic. Firstly, both arsenic samples were dispersed by ultrasonication (10 minutes, 400 W) and subsequently milled at 16 000 rpm for 2 hours with continuous purging of argon. The prepared suspensions were stored in a glovebox under an argon atmosphere.

### Scanning electron microscopy and energy dispersive spectroscopy

The morphology of the material was investigated *via* a SEM with a field emission gun electron source (Tescan Lyra dual microscope) and elemental composition and maps of the materials were obtained using an EDS analyzer (X-Max^N^) with a 20 mm^2^ SDD detector (Oxford Instruments) and AZtecEnergy software. The samples were dropcast on a gold plate after being mixed in an acetonitrile solution. SEM and EDS measurements were carried out using an electron beam in the range of 5–15 kV.

### Transmission electron microscopy

High-resolution TEM was performed with an EFTEM Jeol 2200 FS microscope (Jeol, Japan). A 200 keV acceleration voltage was used for measurement. Elemental maps were acquired with a SDD detector X-MaxN 80 TS (Oxford Instruments, England). All samples prior to the measurements were put on a TEM grid (Cu; 200 mesh; Formvar/carbon).

### X-ray photoelectron spectroscopy

XPS measurements were performed using an ESCAProbeP spectrometer (Omicron Nanotechnology Ltd, Germany) employing a monochromatic aluminum X-ray radiation source (1486.7 eV). Wide-scan surveys of all elements were performed with subsequent high-resolution scans of arsenic (As 3d). The samples were placed on a conductive carrier made from a high purity silver bar. For elimination of the sample charging during measurement an electron gun was utilized (1–5 V). All the XPS survey spectra were afterwards analyzed by CasaXPS software.

### Atomic force microscopy

The AFM measurements were carried out on an Ntegra Spectra from NT-MDT. The surface scans were performed in a tapping (semi-contact) mode. Cantilevers with a strain constant of 1.5 kN m^−1^ equipped with a standard silicon tip with a curvature radius lower than 10 nm were used for all measurements. For the measurements the sample suspension (1 mg mL^−1^) was drop-cast on a freshly cleaved mica substrate. The measurements were performed under ambient conditions with a scan rate of 1 Hz and a scan line of 512.

### X-ray diffraction

XRD patterns were acquired using a Bruker D8 Discoverer powder diffractometer (Bruker, Germany) in Bragg–Brentano parafocusing geometry and applying Cu K_α_ radiation (*λ* = 0.15418 nm and *U* = 40 kV, *I* = 40 mA). The diffraction patterns were collected between 5° and 90° of 2*θ* with a step size of 0.020° and the acquired data were evaluated using HighScore Plus 3.0e.

### Raman spectroscopy

The Raman spectra were taken using an inVia Raman microscope (Renishaw, England) in backscattering geometry with a CCD detector and a DPSS laser (532 nm, 50 mW) with an applied power of 5 mW and a 50× magnification objective. A small amount of each of the samples was placed on a piece of silicon wafer.

### Density functional theory (DFT)

DFT calculations were performed using the projector-augmented wave method as implemented in the Vienna *ab initio* simulation package (VASP).^39,40^ The energy cut-off for the plane-wave expansion was set to 300 eV. The optimized van der Waals optB86b-vdW functional^41,42^ was used because it provided accurate structural properties of various materials combining string intralayer interactions including BP.^43–45^ The calculated lattice parameters were *a* = 3.74, *b* = 4.46 and *c* = 11.02 Å, in excellent agreement with the available experimental data. A dense *k*-point sampling of 16 × 16 × 16 for the A7 structure of g-As and 12 × 10 × 6 mesh for orthorhombic b-As were employed. The elements of the force-constant matrix for the calculation of vibrational modes were determined using density functional perturbation theory. Each mode's off-resonance Raman activity was calculated by evaluating the derivative of the polarizability (or macroscopic dielectric tensor) with respect to the pertinent mode coordinate using the *Raman-sc* package. The symmetry analysis of an irreducible representation of active modes was performed using the *Phonopy* package.^46^

### Fourier transform-infrared spectroscopy

FT-IR measurements were performed on an iS50R FTIR spectrometer (Thermo Scientific, USA). The measurement was performed with a diamond ATR crystal, a DLaTGS detector and a KBr beamsplitter in the range 4000–400 cm^−1^ at a resolution of 4 cm^−1^. The measurement was performed in reflectance mode using a Smart SAGA reflectance accessory and a MCTD* detector and a KBr beamsplitter. The sample for measurement was prepared by drying the dispersion on a gold coated silicon substrate. The measurement of transmittance in the NIR region was performed with a CaF_2_ beamsplitter and with a liquid nitrogen cooled MCT-D* detector.

### Photothermic effect

From As nanoparticles, suspensions were prepared in water resulting in 0.1, 0.25 and 1 mg mL^−1^ concentrations of As. Additionally, 200 μL of Tween was added to suppress the sedimentation of nanoparticles. Before measurement, the tested suspension was sonicated for 5 min. Subsequently, the suspension was irradiated using a laser diode of 880 nm (0.8 W cm^−2^) as biological tissues are most transparent in the 800–900 nm spectral band^47^ and the temperature was recorded using a Pt-100 resistive sensor.

### Electrochemical impedance spectroscopy (EIS)

The characterization by EIS was performed using an Autolab PGSTAT 204 (Metroohm, Switzerland) equipped with a FRA 32M impedance module. Gold interdigitated electrodes (4 μm lines and spacing) were used for the impedimetric electrochemical detection of volatile organic compounds. A sinusoidal potential modulation with an amplitude of 10 mV at a potential of 0.0 V was used to achieve linearity conditions. Applied frequency ranged from 1 MHz to 1 Hz with 10 measured points per decade.

**Fig. 2:**
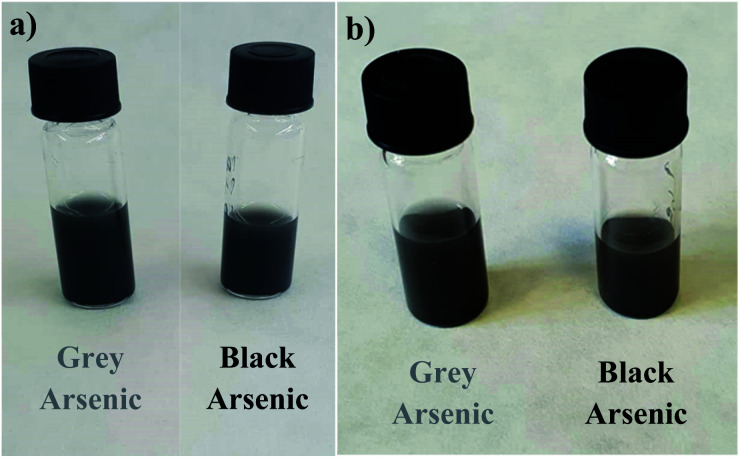
Suspensions of exfoliated grey (left) and black (right) arsenic in acetonitrile (a) after 1 hour and (b) after 8 days.

The samples were dispersed in acetonitrile to obtain a 2 mg mL^−1^ suspension (Fig. 2a). The suspension was sonicated for 5 min at room temperature before the use. An interdigitated electrode was modified by drop-casting of a 50 μL aliquot of the suspension which was dried for 15 min in a vacuum oven at 55 °C to obtain a layer of randomly dispersed materials on the electrode surface. The electrodes were tested by cyclic voltammetry to verify conductivity of the modified electrodes and to achieve linearity conditions prior to the EIS measurements. The modified electrodes were left in a vacuum oven for 15 min before every single EIS measurement. A 50 mL flask was used as the electrochemical cell. The flask was filled with 50 μL of the investigated compound, sealed and left to equilibrate for 120 s to achieve steady state conditions.

## Results and discussion

### Characterization of materials

The phase purity of the starting materials was identified by X-ray diffraction confirming the rhombohedral and orthorhombic structures for grey and black arsenic, respectively (PDF 04-003-6953 and 03-065-2495) (Fig. S1†). These arsenic allotropes were exfoliated in acetonitrile by high-energy shear force milling and the prepared suspensions of single/few layer of each respective material were used for further characterization and application exploration. Both materials had a stable dispersion for 8 days as pure acetonitrile is capable of effectively stabilizing exfoliated arsenene^37^ (Fig. 2b). The process of the exposure of exfoliated arsenic materials to ambient conditions is shown in Fig. S2† where their stability after 8 days and degradation after 9 days are shown. The degradation of the suspensions after 9 days can be observed from the clear sedimentation of the material and the solution became more transparent.

The morphology of bulk grey and black arsenic used for exfoliation is shown in Fig. S3.† On both materials a layered structure is clearly observed with small pieces due to the materials having been mechanically ground prior to the measurements.

After the exfoliation of both materials in acetonitrile, AFM images were acquired to observe the thickness. In Fig. S4,† the images accompanied by the measured height profile are depicted. It becomes evident that the exfoliation procedure for b-As is more efficient producing arsenic nanosheets with a thickness below 3 nm in comparison to the g-As nanosheets possessing a thickness between 20 and 30 nm. Despite the approximately similar bond lengths of arsenic atoms in both materials, the distance between layers is larger in b-As (Table S1†). This is caused by the orthorhombic nature of the b-As structure as the layers are held together by weak van der Waals forces in contrast to the rhombohedral structure of g-As, where the layers are held together more tightly.

**Fig. 3:**
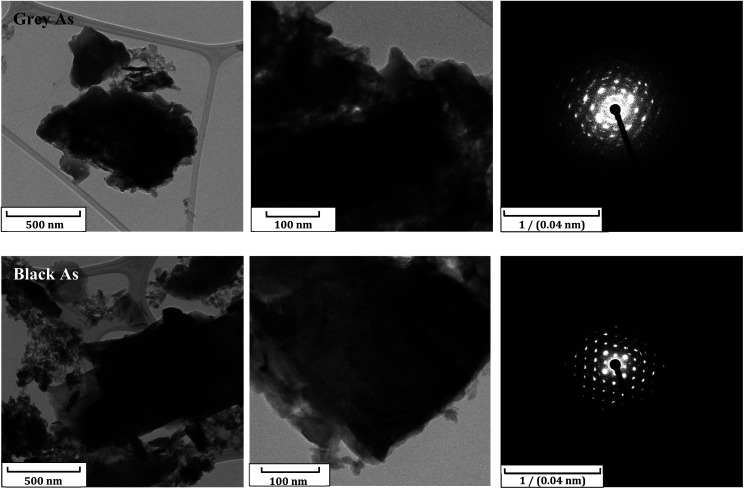
TEM/SAED of grey and black arsenic nanoparticles.

Additional characterization was carried out through TEM combined with selected area electron diffraction (SAED) and EDS. TEM images revealed a sheet structure for both materials and the SAED patterns of sheets showed the rhombohedral and orthorhombic structures for grey and black arsenic, respectively (Fig. 3). The SEM/EDS and TEM/EDS images exposed the uniform presence of arsenic over the sheets including the presence of oxygen in both materials (Fig. S5 and S6†). Nevertheless, since the distribution does not resemble the As elemental maps, it was concluded that the presence of oxygen is due to atmospheric contamination and that possible oxide formation is local. Therefore, according to the EDS elemental maps from TEM we have confirmed the existence of few-layered arsenene and excluded the formation of arsenic oxide on the exfoliated sheets.

The surface composition of exfoliated black and grey arsenic was examined by XPS, a useful technique to examine the oxidation state of arsenic within a material. The wide-survey spectra confirmed the presence of arsenic in both materials (Fig. S7†). Adventitious carbon is also present along with oxygen originating from either atmospheric contamination or arsenic oxide (As_2_O_3_) formation on the surface of the materials.

**Fig. 4:**
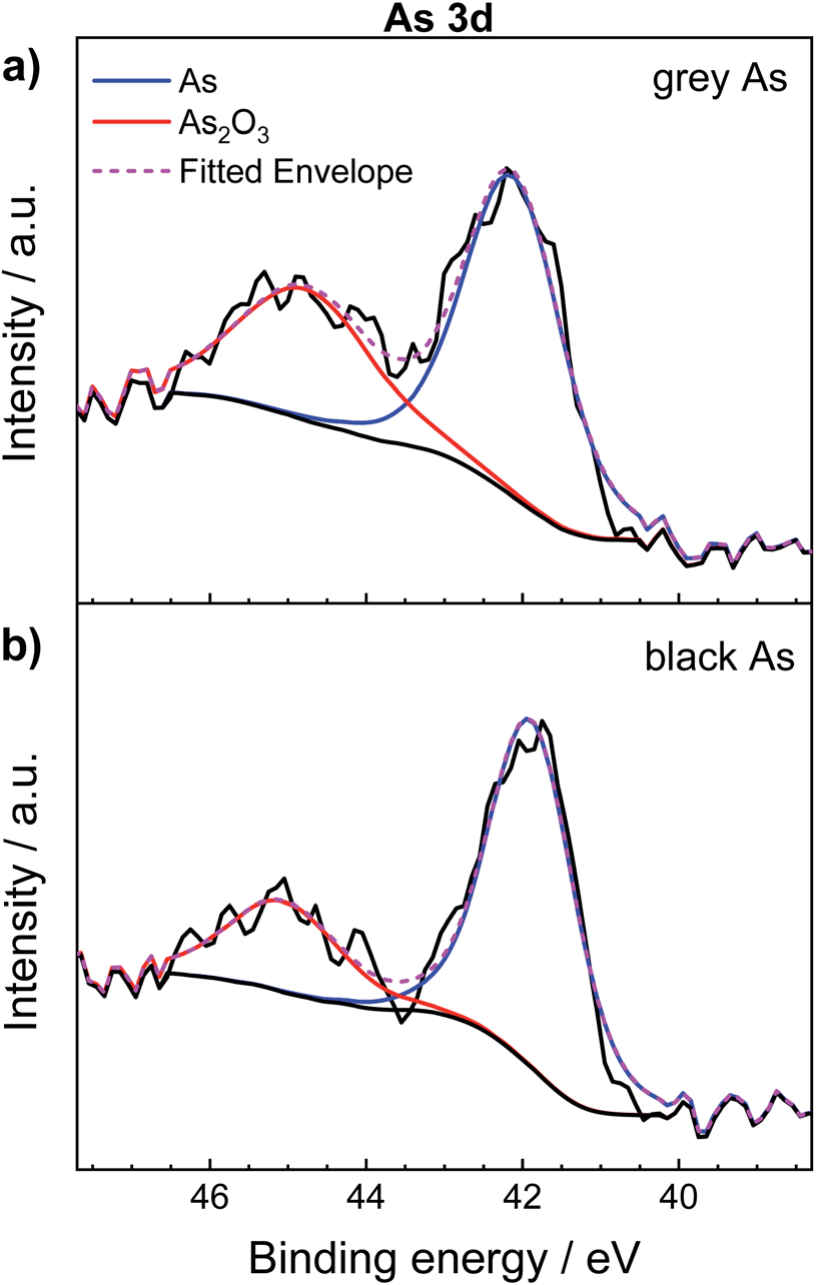
High-resolution As 3d XPS spectra of (a) grey arsenic and (b) black arsenic.

In addition, Fig. 4 shows the comparison of the high-resolution XPS spectrum of As 3d between exfoliated grey and black arsenic. Two distinguishable peaks clearly formed for both materials. The sharp peak at 42.2 eV corresponds to the As–As binding energy of elemental arsenic^22,48^ while the signal at 44.9 eV is due to the inevitable formation of As_2_O_3_ on the material surface caused by the atmospheric oxygen. According to the XPS results, it is likely that black arsenic is less susceptible to oxidation as evidenced by the calculated As : As_2_O_3_ ratio presented in Table 1. However, it is a well-known fact that arsenic trioxide sublimes in a vacuum^49^ and that the previous results demonstrate the high tendency of arsenic towards oxidation. This effect is also well known from the surface chemistry of black phosphorus, which undergoes rapid formation of phosphorus oxides on the surface.^50^

**Table 1:** Calculation of the ratio between elemental arsenic and arsenic oxide after analysis of the high-resolution As 3d XPS spectra

Material	%As	%As_2_O_3_	As/As^3+^ ratio
Grey arsenic	62.70	37.30	1.68
Black arsenic	73.59	26.41	2.78

**Fig. 5:**
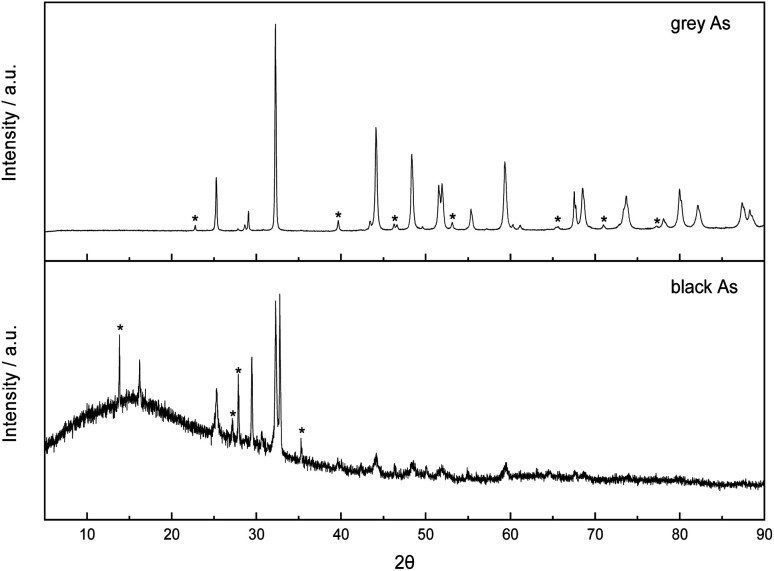
Comparison of X-ray diffraction patterns of exfoliated grey and black arsenic. Peaks marked with an asterisk are due to the presence of arsenic oxide in the sample.

Both exfoliated allotropes of arsenic were further explored by X-ray diffraction (Fig. 5). Firstly, the rhombohedral *R*3̄*m* crystal structure of grey arsenic (PDF 01-073-5919) was confirmed with the most intense diffraction pattern at 2*θ* = 32.2° corresponding to the (012) reflection. Some minor peak diffraction pattern noticeable in the XRD pattern had arisen due to the presence of As_2_O_3_.

Regarding the XRD pattern of the exfoliated black arsenic, the non-flat baseline is indicative of the presence of amorphous arsenic also visible in the SEM images. The most prominent feature is a sharp doublet peak at 2*θ* of 32.55 and 32.80° characteristic of the allotropic orthorhombic crystal structure of arsenic (PDF 00-029-0142) and corresponding to the (004) and (111) phases, respectively. However, the presence of arsenic oxide is much more pronounced in the black As XRD pattern where the relevant peaks are more intense in comparison to the pattern of grey As and there were also some minor peaks arising due to rhombohedral arsenic. Finally, the sharp narrow peaks in both patterns revealed the high crystallinity of the materials.

**Fig. 6:**
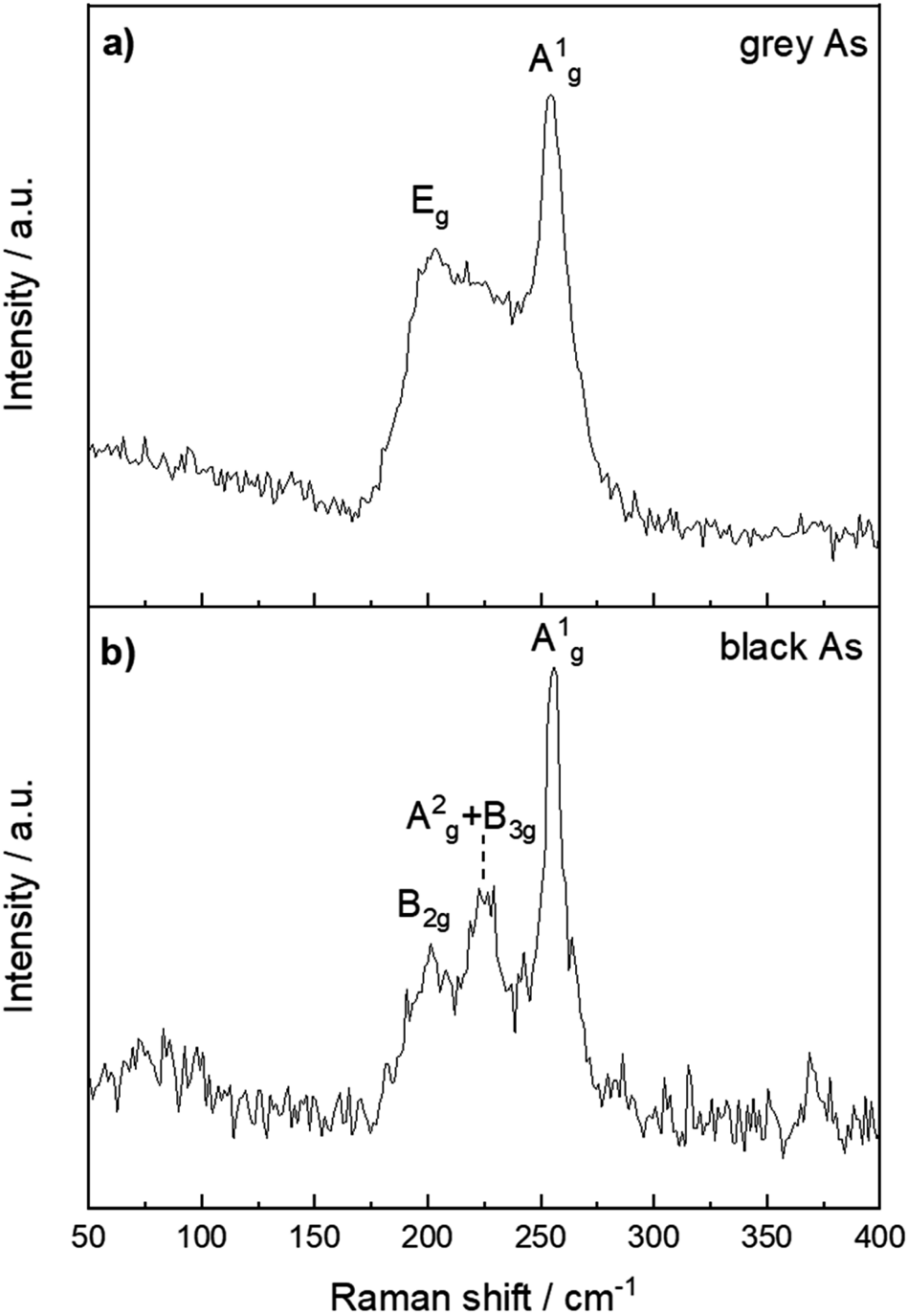
Raman spectra of (a) black arsenic and (b) grey arsenic with their respective phonon modes labelled.

The structural differences between grey and black arsenic were further examined by Raman spectroscopy and depicted in Fig. 6. The A7 structure of g-As belongs to the *R*3̄*m* space group which has three Raman active modes, a two-fold degenerate E_g_ and A^1^_g_. The modes are clearly visible at 202 and 254 cm^−1^.^51^ The DFT calculations confirm the presence of two active modes where the calculated wavenumbers of the modes are 184 and 245 cm^−1^. For vibrations perpendicular to the layers of the rhombohedral lattice, the A^1^_g_ mode belongs to a pure longitudinal motion of the atom planes, whereas two E_g_ modes correspond to transverse motions.

The structure of b-As has the *Cmca* (no. 64) space group and there are 12 lattice vibrational modes at the G point. According to the character table, six of them should be Raman active with the irreducible representations 2A_g_ + B_1g_ + B_2g_ + 2B_3g_. The Raman spectrum in Fig. 6b nevertheless reveals three distinct peaks at 201, 223 and 255 cm^−1^. Three phonon modes were observed also in an earlier study of b-As as well^35^ at slightly shifted frequencies (224, 230 and 258 cm^−1^) and possess similar features to the well-indexed Raman spectrum of black phosphorus.^52^ Our DFT calculations yield six Raman active modes at 90, 104, 206, 216, 228 and 239 cm^−1^, in agreement with the symmetry considerations above. For an irreducible representation of each mode, we assign them to the out-of-plane B_1g_, B_3g_, B_2g_, A^2^_g_ and in-plane B_3g_ and A^1^_g_ in the respective order. The less intense modes at 90 and 104 cm^−1^ are not visible in the spectrum due to the background noise. The A^2^_g_ and B_3g_ modes at 216 and 228 cm^−1^ are most likely convoluted in the Raman spectrum and give rise to a broad middle peak. Thus, the three peaks in the spectrum confirm the orthorhombic crystal structure of the material. It should be noted that owing to the anisotropic character of the structure. The relative intensity of the mode's Raman shifts depends on the sample orientation with respect to the direction and polarization of the incident laser light.^35,53^

### Photothermic effect

**Fig. 7:**
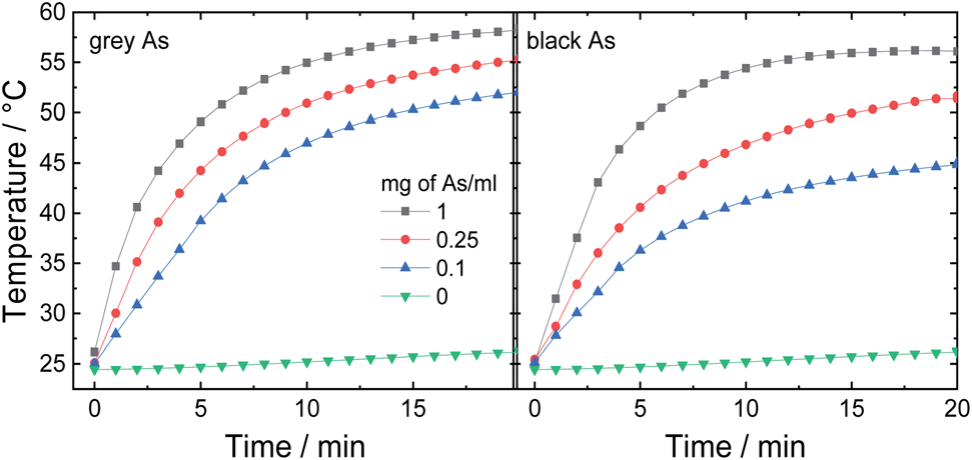
Photothermic effect measured on three different As concentrations using an 880 nm (0.8 W cm^−2^).

In the next step, potential applications of arsenic nanoparticles were explored. Firstly, the photothermic effect was evaluated using an 880 nm laser irradiation. Three different arsenic concentrations were tested and pure water for comparison (Fig. 7). While pure water exhibited only a negligible increase in temperature, all arsenic suspensions showed fast elevation during the first 5 minutes and afterwards, the temperature was gradually increasing until it reached a maximum temperature plateau. After 20 minutes, black As reached final temperatures of 45, 51 and 56 °C and grey As reached 52, 55 and 58 °C for As concentrations of 0.1, 0.25 and 1 mg mL^−1^, respectively. Thus, both samples exhibited a similar photothermic effect.

### Gas sensing

Lastly, the potential application of the prepared samples as impedimetric sensors for volatile organic compounds (VOCs) was investigated. Both arsenic samples were dropcast on gold interdigitated electrodes. Between the gold electrode and the active material, two types of contacts can exist – ohmic or Schottky. First of all, a cyclic voltammogram was obtained in the range between −1.5 and 1.5 V to distinguish which type of transition is present (Fig. S8†). Grey As exhibited a linear current–voltage (*I*–*V*) curve in the whole potential range due to its metallic character thus this sensor exhibits an ohmic transition. On the other hand, black As exhibited Schottky transition and has a nonlinear *I*–*V* curve.

**Fig. 8:**
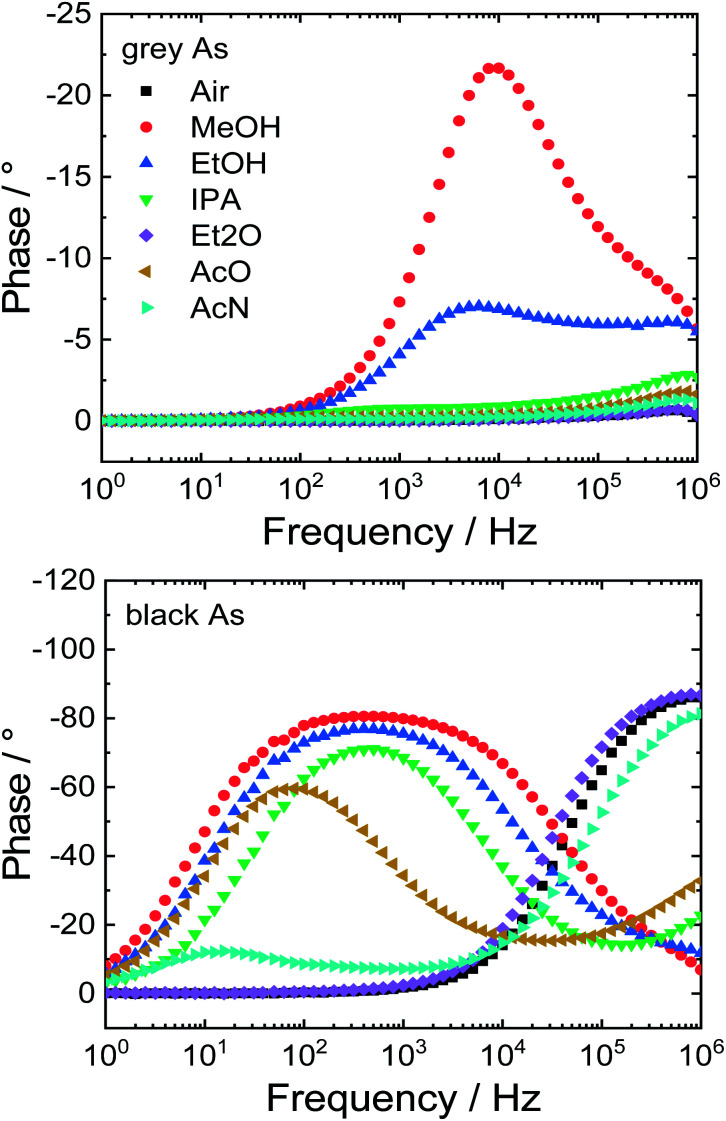
Response of impedimetric sensors to various VOCs plotted as Bode diagrams.

Following this, the modified interdigitated electrodes were exposed to various VOCs and ambient air for comparison and the impedimetric responses were collected. These responses are displayed as Bode and Nyquist plots (Fig. 8 and S9†, respectively). The Bode plot shows frequency dependence of phase shift which can be utilized to differentiate between individual VOCs. Grey As showed sensitivity towards two VOCs: methanol and ethanol with maxima of phase shift at 9 (−21°) and 6 kHz (−7°), respectively. Black As was sensitive towards five VOCs: methanol, ethanol isopropanol, acetone and acetonitrile with maxima of phase shift at 400 (−80°), 400 (−77°), 500 (−71°), 80 (−60°) and 13 (−12°) Hz, respectively. Moreover, the stability of the impedimetric sensors was evaluated by a repetition of measurements in ambient air after the exposure to the tested VOCs (Fig. S10†). Only slight shifts of the measured points were observed that confirmed the good stability of the tested impedimetric sensors.

## Conclusion

In summary, compelling experimental evidence has been demonstrated for a novel exfoliation method of grey and black arsenic nanoparticles. SEM images revealed the existence of ordered stacked layers for both arsenic samples while structural characterization confirmed the orthorhombic and rhombohedral structures of black and grey arsenic before and after exfoliation. Additionally, both exfoliated samples were tested for potential applications in photothermic absorption and as impedimetric sensors of VOCs. While both samples exhibited a similar photothermic effect, exfoliated black arsenic was revealed to be more suitable as an impedimetric VOC sensor.

## Conflicts of interest

The authors do not have any conflicts of interest.

## Supplementary Material

NA-002-C9NA00754G-s001
