# Supplementary material for: Acetonitrile-assisted exfoliation of layered grey and black arsenic: contrasting properties
Source: Nanoscale Adv. 2020 Feb 3;2(3):1282–9. doi: 10.1039/c9na00754g (PMC9417731; doi:10.1039/c9na00754g)
Supplement: NA-002-C9NA00754G-s001 [file NA-002-C9NA00754G-s001.pdf]

Electronic Supplementary Material (ESI) for Nanoscale Advances.
This journal is © The Royal Society of Chemistry 2020

SUPPORTING INFORMATION

Acetonitrile-Assisted Exfoliation of Layered Grey and Black Arsenic: Contrasting Properties

Nikolas Antonatos[1], Vlastimil Mazánek[1], Petr Lazar[2], Jiri Sturala[1], Zdeněk Sofer[1,]*

[1]Department of Inorganic Chemistry, University of Chemistry and Technology Prague, Technická 5, 166 28 Prague 6, Czech Republic

[2]Regional Centre of Advanced Technologies and Materials, Faculty of Science, Palacký University Olomouc, tř. 17. listopadu 12, 77 146 Olomouc, Czech Republic.

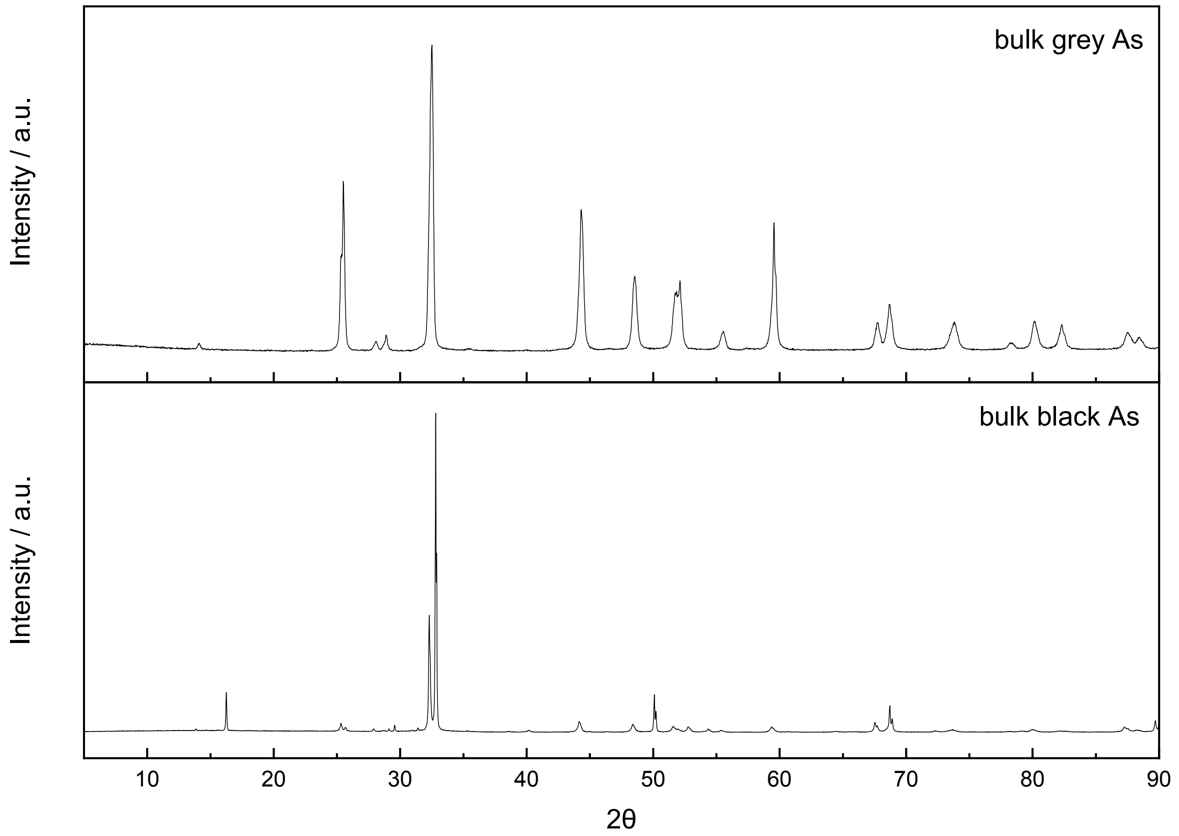

Figure S1. Comparison of X-ray diffraction patterns of bulk grey and black arsenic.

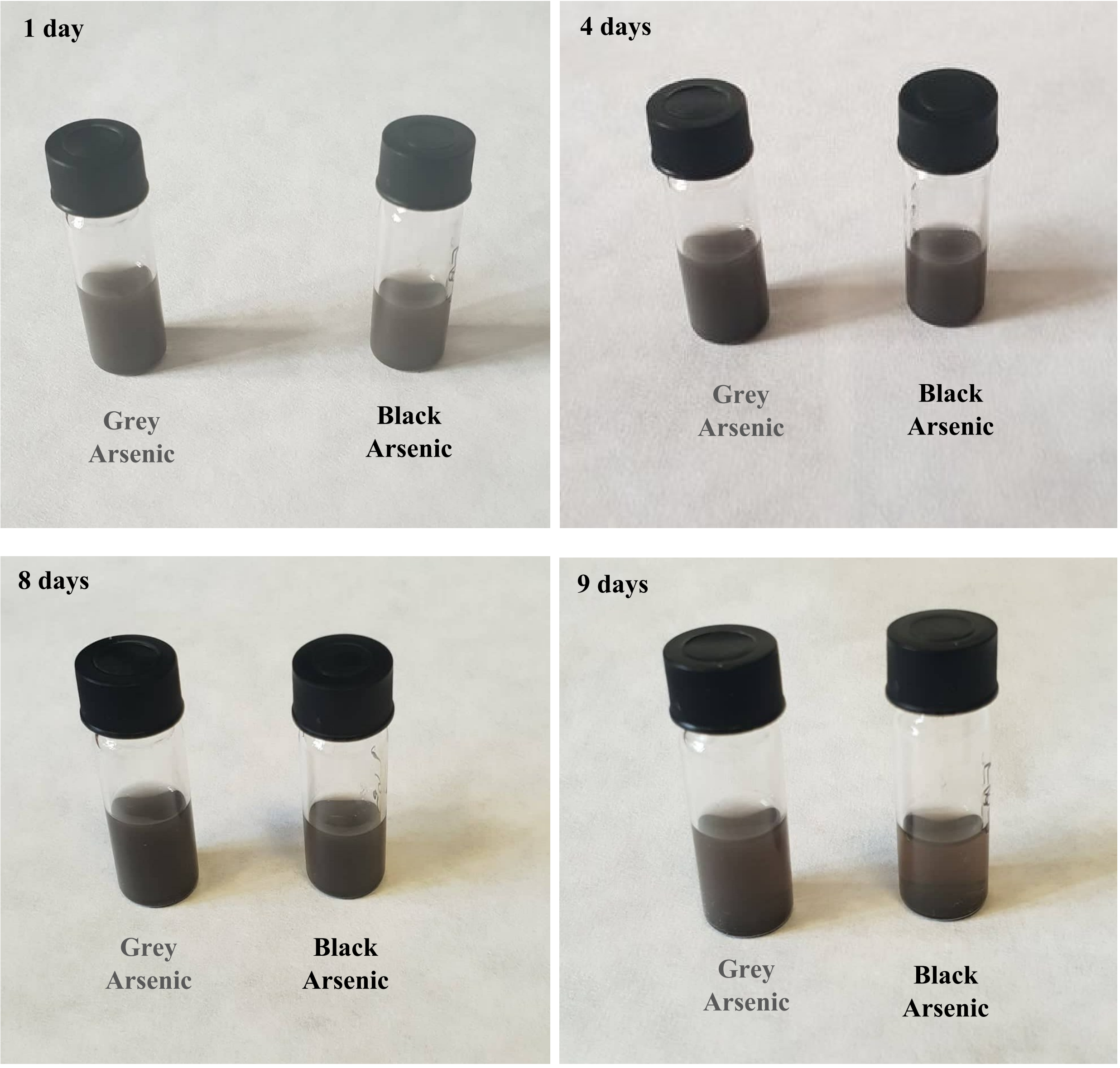

Figure S2. Suspensions of exfoliated grey and black arsenic in acetonitrile after 1, 4, 8 and 9 days depicting the ability of acetonitrile to stabilize the dispersions in ambient conditions for a long time (8 days).

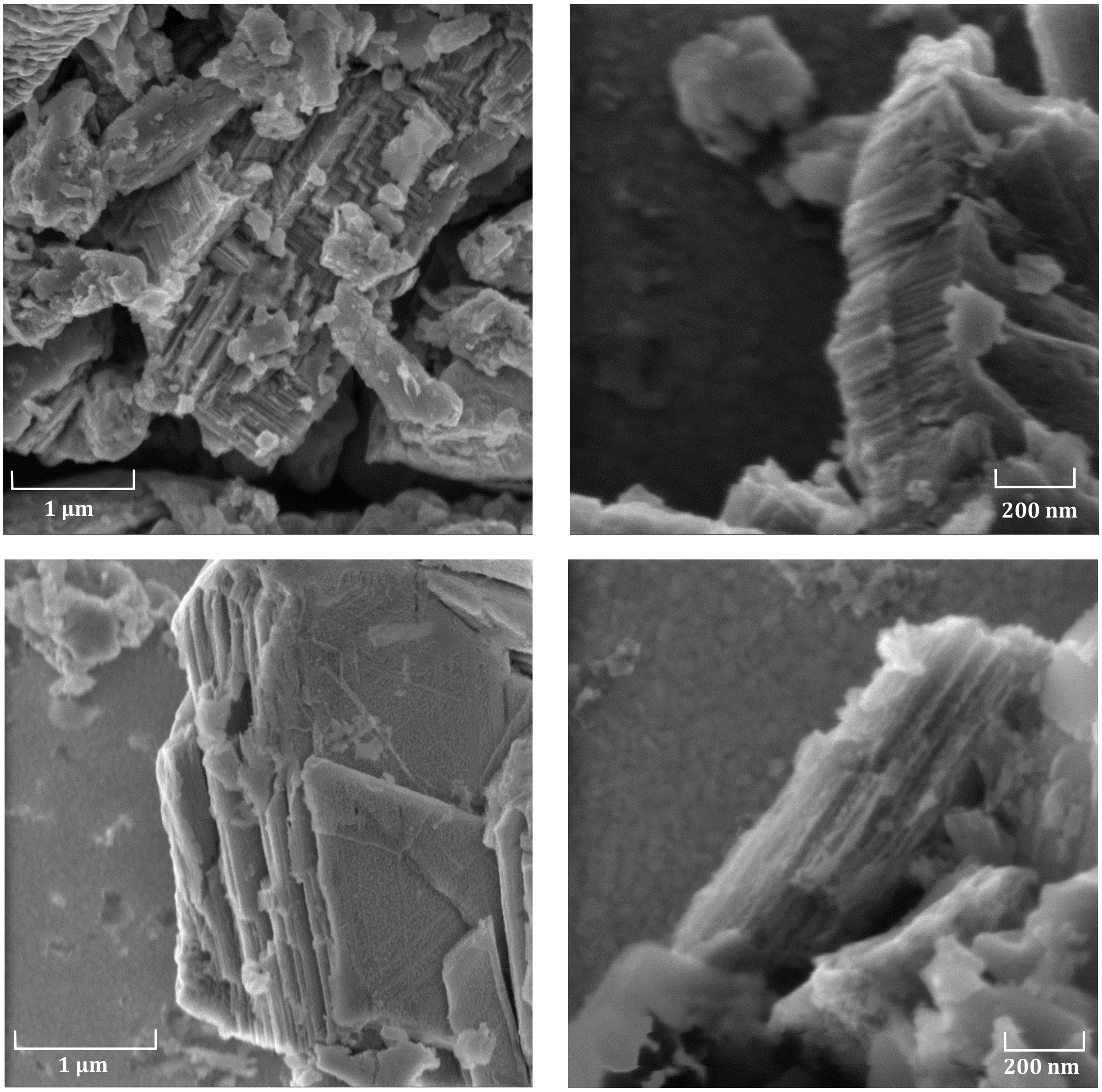

Figure S3. SEM images of bulk grey arsenic (**top**) and black arsenic (**bottom**)

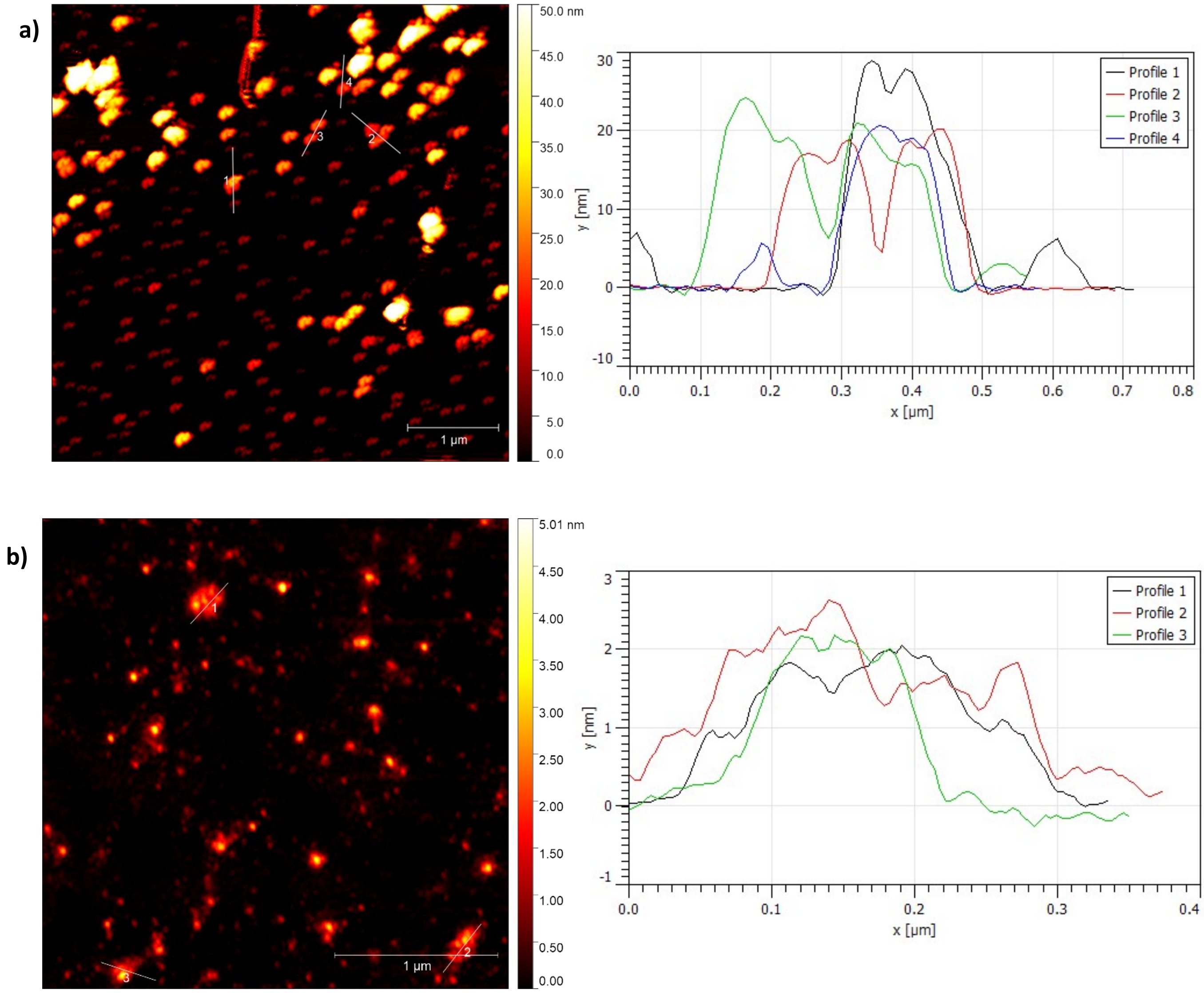

Figure S4. AFM images with corresponding height profiles of **a)** grey arsenic and **b)** black arsenic.

Table S1. Comparison of bond lengths between atoms and distance between layers for grey and black arsenic.

Material	As-As bond length / Å		Interlayer distance / Å
	Same plane	Different plane	
g-As[1]	2.50	2.53	3.52
b-As[2]	2.50	2.48	5.46

[1] Z. Zhu, J. Guan, D. Tománek, *Phys. Rev. B,* 2015, **91,** 161404(R)
[2] Y. Chen, C. Chen, R. Kealhofer, H. Liu, Z. Yuan, L. Jiang, J. Suh, J. Park, C. Ko, H.S. Choe, J. Avila, M. Zhong, Z. Wei, J. Li, H. Gao, Y. Liu, J. Analytis, Q. Xia, M.C. Asensio and J. Wu, *Adv. Mater.,* 2018, **30,** 1800754

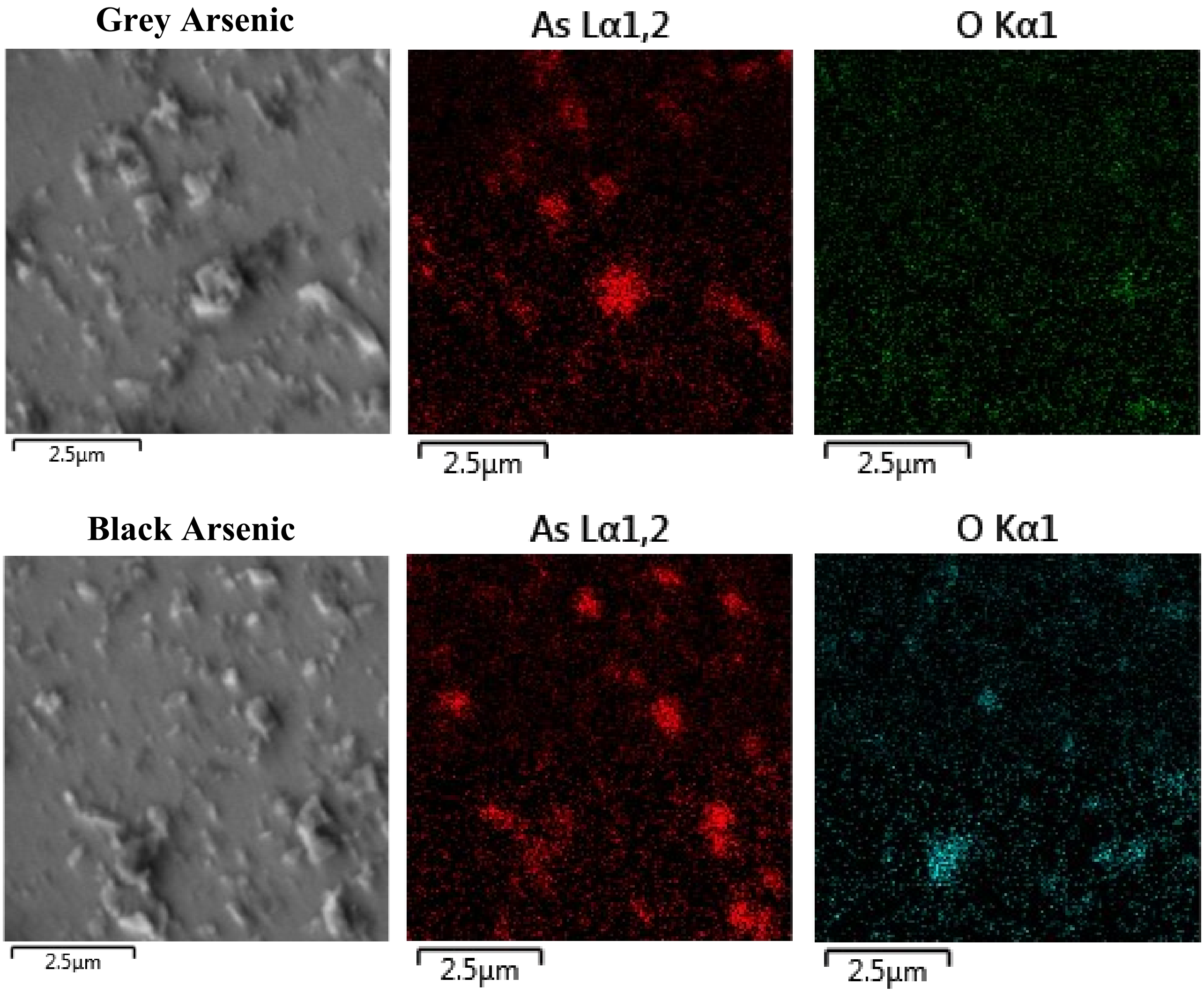

Figure S5. SEM images of exfoliated grey (**top**) and black (**bottom**) arsenic with their corresponding EDS elemental maps of arsenic and oxygen.

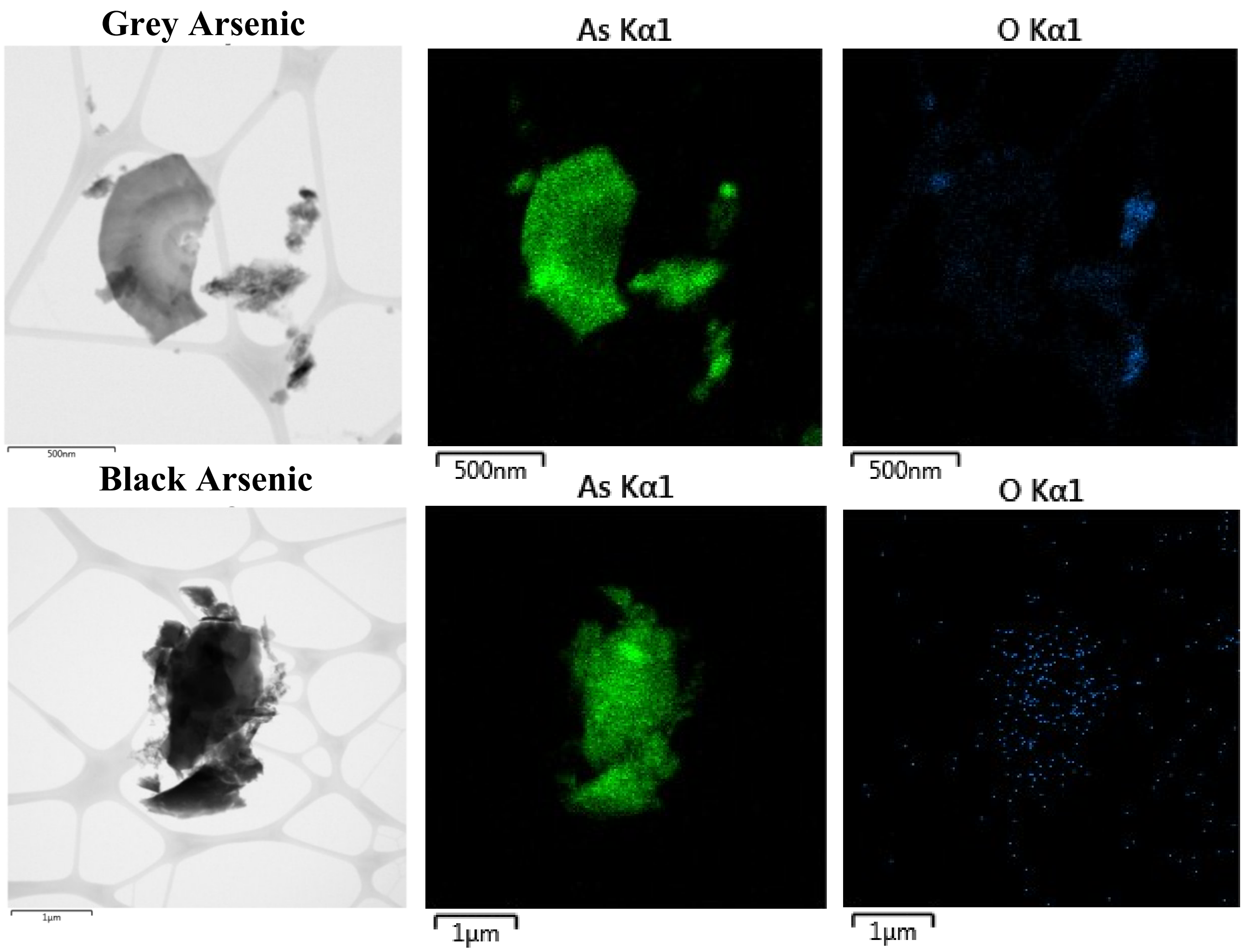

Figure S6. TEM images of exfoliated grey (**top**) and black (**bottom**) arsenic with their corresponding EDS elemental maps of arsenic and oxygen.

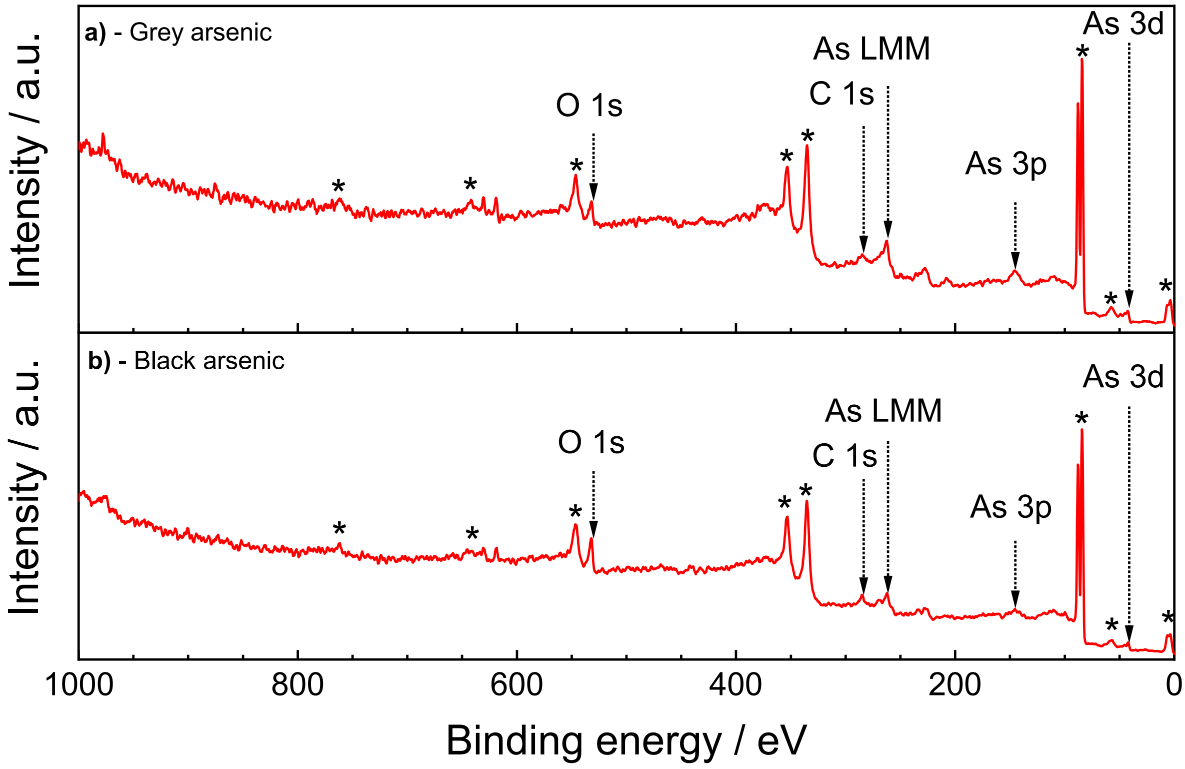

Figure S7. Wide survey XPS spectra of **a)** grey and **b)** black arsenic. Marked with an asterisk are signals caused by the Au substrate.

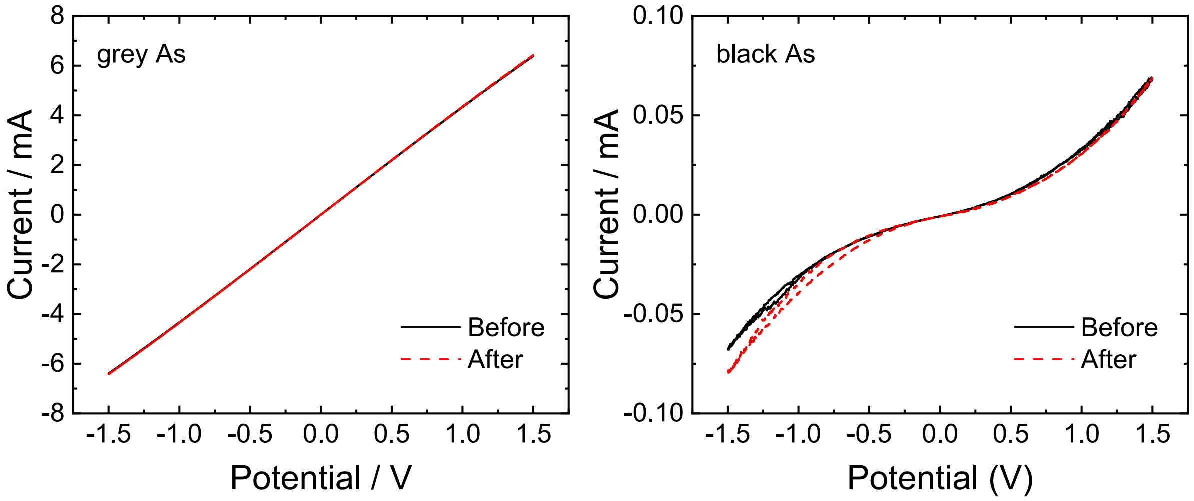

Figure S8. I-V curves of modified impedimetric sensors of grey and black arsenic measured before (**black**) and after (**red**) exposure to tested VOCs.

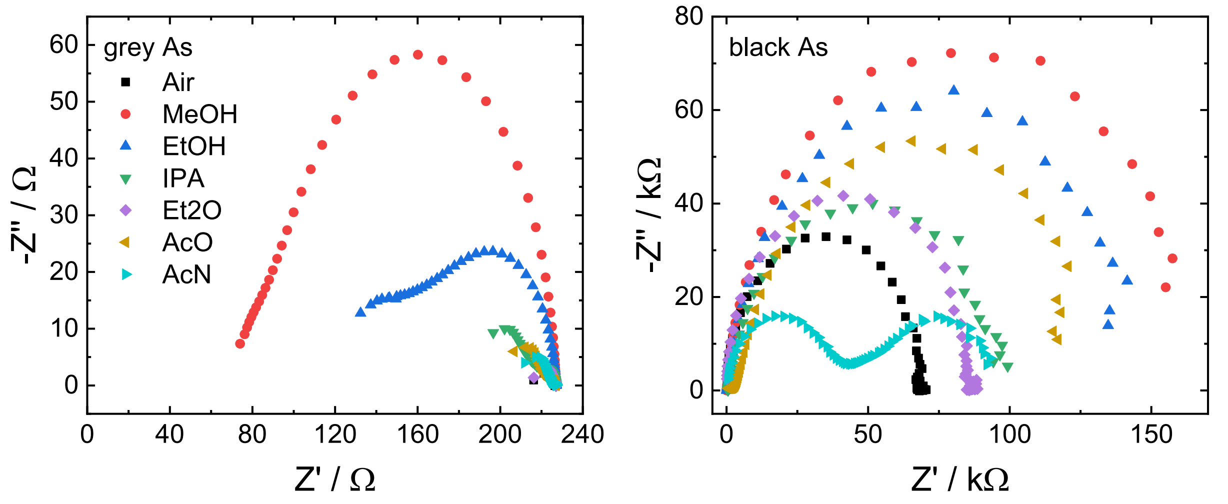

Figure S9. Response of impedimetric sensors to various VOCs plotted as Nyquist diagrams.

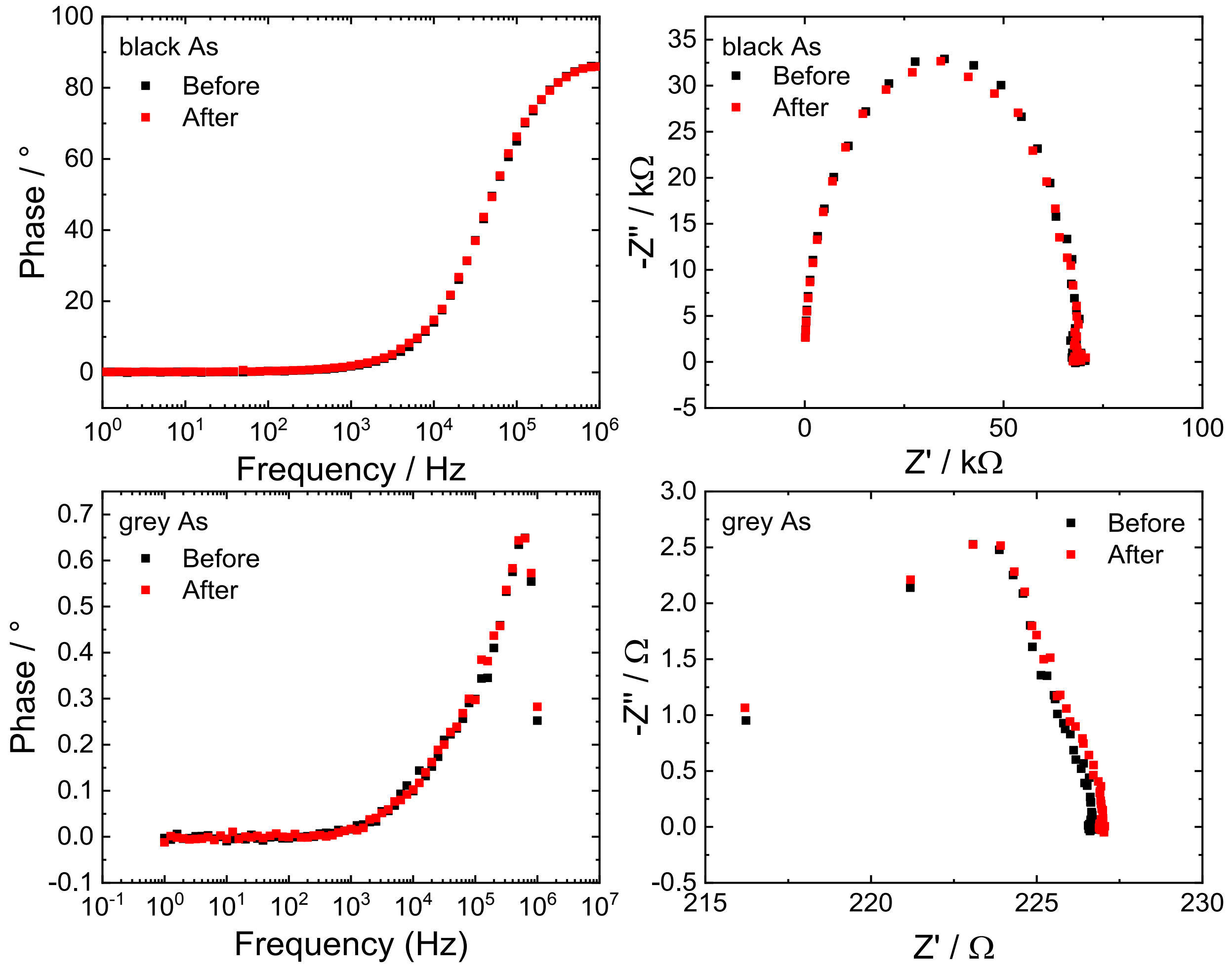

Figure S10. Response of the sensors before (**black**) and after (**red**) exposure to all tested VOCs. Left hand graphs are Bode diagrams and Nyquist diagrams are in the right.
